# Chronic NaHS Treatment Is Vasoprotective in High-Fat-Fed ApoE^−/−^ Mice

**DOI:** 10.1155/2013/915983

**Published:** 2013-06-24

**Authors:** Asha Ford, Mohammad Al-Magableh, Tracey A. Gaspari, Joanne L. Hart

**Affiliations:** ^1^School of Medical Sciences and Health Innovations Research Institute, RMIT University, Bundoora, VIC 3083, Australia; ^2^Department of Pharmacology, Monash University, Clayton, VIC 3800, Australia

## Abstract

Hydrogen sulfide is emerging as an important mediator of vascular function that has antioxidant and cytoprotective effects. The aim of this study was to investigate the role of endogenous H_2_S and the effect of chronic exogenous H_2_S treatment on vascular function during the progression of atherosclerotic disease. ApoE^−/−^ mice were fed a high-fat diet for 16 weeks and treated with the H_2_S donor NaHS or the cystathionine-**γ**-lyase (CSE) inhibitor D,L-propargylglycine (PPG), to inhibit endogenous H_2_S production for the final 4 weeks. Fat-fed ApoE^−/−^ mice displayed significant aortic atherosclerotic lesions and significantly impaired endothelial function compared to wild-type mice. Importantly, 4 weeks of NaHS treatment significantly reduced vascular dysfunction and inhibited vascular superoxide generation. NaHS treatment significantly reduced the area of aortic atherosclerotic lesions and attenuated systolic blood pressure. Interestingly, inhibiting endogenous, CSE-dependent H_2_S production with PPG did not exacerbate the deleterious vascular changes seen in the untreated fat-fed ApoE^−/−^ mice. The results indicate NaHS can improve vascular function by reducing vascular superoxide generation and impairing atherosclerotic lesion development. Endogenous H_2_S production via CSE is insufficient to counter the atherogenic effects seen in this model; however exogenous H_2_S treatment has a significant vasoprotective effect.

## 1. Introduction

Hydrogen sulfide is a recently identified gasotransmitter reported to have numerous physiological effects in diverse processes including metabolism, inflammation, the nervous system, and the cardiovascular system [1]. The cardiovascular effects of this molecule are currently of major interest and include vascular relaxation, cardioprotection, and vasculoprotective effects [2, 3].

In mammalian cells, H_2_S is produced primarily by 2 pyridoxyl-5′-phosphate-dependent enzymes, cystathionine-*β*-synthase (CBS), and cystathionine-*γ*-lyase (CSE). Additionally, a role for 3-mercaptopyruvate sulfurtransferase in concert with cysteine aminotransferase has been identified in the vasculature [4]. With respect to vasoregulation, CSE is of particular interest as it is reported to be present in a range of vascular beds and its expression has been clearly identified in vascular smooth muscle cells. CSE has also been located in endothelial cells and additionally it is reported to contribute to endothelium-dependent vasorelaxation [5, 6]. Inhibition of CSE with the irreversible inhibitor D,L-propargylglycine (PPG) leads to an elevation of blood pressure *in vivo* [7] and increased vascular tone *in vitro* [8]. Importantly, mice deficient in CSE are hypertensive compared to their wild-type controls [5]. Collectively, these reports point to a role for H_2_S in the regulation of vascular function.

Atherosclerosis is the most common underlying cause in the development of coronary artery disease, a leading cause of death and morbidity worldwide. Atherosclerosis is a chronic immunoinflammatory, fibroproliferative disease caused by lipid accumulation, affecting large- and medium-sized arteries [9, 10]. Atherosclerosis has a multifactorial pathogenesis, involving vascular inflammation, including increased reactive oxygen species generation which leads to impairment of vascular endothelial function, by reducing NO bioavailability [11].

A number of previous studies have indicated that H_2_S has many properties that may lead to the inhibition of atherogenesis (for review see [12]). These properties include inhibition of proliferation [13], induction of apoptosis [14] in vascular cells, inhibition of oxidative damage, and decreased production of reactive oxygen species (ROS) [15, 16]. These effects are all cytoprotective, and additionally H_2_S treatment leads to decreased foam cell production [17], a reduction in adhesion molecule expression [18] decreased chemokine signalling [19] and decreased platelet aggregation [20]. H_2_S is also reported to have antiinflammatory [21] and anti-remodelling effects [22], in addition to vasorelaxant effects in both large [8, 23–25] and small [26–28] blood vessels which could also be beneficial in preventing the progression of vascular disease.

This study focuses on the anti-oxidant properties of H_2_S that suggest that it may be a useful agent in the treatment or prevention of vascular dysfunction in atherosclerosis. Thus, our hypothesis was that H_2_S will act as a vascular protective factor, due to inhibitory effects on superoxide production and action in the vasculature. The specific aims of this study were to examine the role of both endogenously produced H_2_S and chronic exogenous H_2_S treatment *in vivo* on a lesion development, vascular superoxide generation, and endothelial function in the fat-fed ApoE^−/−^ mouse model of atherosclerosis. 

## 2. Materials and Methods

### 2.1. Animals and Ethics

All experimental procedures involving the use of animals were carried out in accordance with the “Guide for the Care and Use of Laboratory Animals” published by the US National Institutes of Health and the project was approved by the RMIT University Animal Ethics Committee prior to the commencement. All animals were housed in the RMIT Animal Facility, RMIT University, Bundoora West Campus, on a 12-hour day/night cycle at room temperature of 20 ± 2°C. Male ApoE^−/−^ mice with a C57BL/6J background (*n* = 64) and male C57BL/6J wild-type (WT) mice were purchased from the Animal Resource Centre, Western Australia. From 5 weeks of age mice were fed a high-fat diet containing 22% fat, 0.15% cholesterol (Specialty Feeds, Western Australia) for 16 weeks. 

### 2.2. Treatments

During the final four weeks of fat feeding, mice were assigned to 4 groups: (1) untreated, (2) treated with NaHS to deliver H_2_S at 10 *μ*mol/kg/day, (low NaHS), (3) NaHS 100 *μ*mol/kg/day (high NaHS), or (4) D,L-propargylglycine (PPG) 30 mg/kg/day. All treatments were administered daily by intraperitoneal injection. Adult male C57 mice (*n* = 6), aged 16 weeks and fed on standard rodent chow, were also used as a wild-type (WT) control group. 

### 2.3. Systolic Blood Pressure Measurement

Systolic blood pressure was measured at weeks 12 and 16 (before and after treatment), using the noninvasive tail-cuff apparatus (ADInstruments, Sydney). Systolic blood pressure was averaged from four to six consecutive measurements taken at intervals of 1-2 mins. 

### 2.4. Tissue Collection

Mice were culled in a humane manner via CO_2_ asphyxiation (95% CO_2_, 5% O_2_), followed by cervical dislocation and decapitation. The aorta and liver were dissected out and washed in ice-cold oxygenated Krebs' solution (composition in mM: NaCl 119, KCl 4.7, MgSO_4_ 1.17, NaHCO_3_ 25, KH_2_PO_4_ 1.18, CaCl_2_ 2.5, glucose 5.5, EDTA 0.026, and pH 7.4). Aortic arches (for oil red O staining) and abdominal aorta segments (for endothelial function assays) were used immediately. Blood plasma samples were snap frozen in N_2_ for later use.

### 2.5. Plasma Lipid Measurements

Total plasma cholesterol, high-density lipoprotein (HDL), low-density lipoprotein (LDL), and triglyceride levels were measured with a Konelab 20XTi Random Access Analyser.

### 2.6. Measurement of Lesion Formation

Development of atherosclerotic lesions across the aortae was assessed using *en face* staining with Oil Red O. The aortae were dissected free of connective tissue and stained with oil red O then washed in 60% isopropyl alcohol. Longitudinal segments were then photographed with a digital camera. Image J was used for analysis of total tissue to lesion area ratio via manual tracing with measurements based on a calibration image of 1 mm. Lesion area was expressed as a percentage of the total luminal area.

### 2.7. Endothelial Function Assay

Abdominal aortic rings were mounted in myograph chambers, where they were maintained in Krebs' solution at 37°C, continuously supplied with carbogen (95% CO_2_, 5% O_2_). The aortic rings were allowed to equilibrate for 20 min under zero force, then a 5 mM resting tension was applied. Changes in isotonic tone were recorded using Myograph Interface model 610 M (ADInstruments, Sydney) and the myodac data acquisition system. Concentration response curves to the endothelium-dependent dilator acetylcholine (ACh) were constructed in vessels preconstricted with the thromboxane analogue U46619. Precontraction to U46619 was submaximal and not significantly different between groups. At the end of the ACh curve, 10 *μ*M of the endothelium-independent dilator levcromakalim (LKM) was added to test vascular smooth muscle cell function.

### 2.8. Measurement of Vascular Superoxide Generation

Superoxide anion production in the aorta was determined by lucigenin-enhanced chemiluminescence assay as previously described [29, 30]. Briefly, sections of abdominal aorta (3 mm long) were preincubated for 45 min at 37°C in Krebs-HEPES buffer (composition (mM): NaCl 99.9, KCl 4.7, KH_2_PO_4_ 1.0, MgSO_4_·7H_2_O 1.2, D-glucose 11.0, NaHCO_3_ 25.0, CaCl_2_·2H_2_O 2.5, Na HEPES 20.0, and pH 7.4) containing diethyldithiocarbamic acid (1 mM) to inactivate superoxide dismutase and NADPH (100 *μ*M) as a substrate for NADPH oxidase. 300 *μ*L of Krebs-HEPES buffer, containing lucigenin (5 *μ*M) with and without the flavoprotein inhibitor diphenylene iodonium (DPI, 1 *μ*M) to inhibit NADPH oxidase, was placed into a 96-well OptiPlate, which was loaded into a POLARstar Optima photon counter (BMG Labtech, Melbourne, VIC, Australia) to measure background photon emission at 37°C. After background counting was completed, a single ring of aorta was added to each well and photon emission was recounted. The background reading was subtracted from the superoxide anion counts and normalized with dry tissue weight.

### 2.9. Data Analysis

Results are expressed as mean ± standard error of the mean (SEM) with the number of experiments denoted by *n*. Concentration response curves to ACh were expressed as a percentage reversal of the U46619 precontraction. These data were computer fitted to a sigmoidal curve using nonlinear regression (GraphPad Prism, version 5.0) to calculate the sensitivity of the vasorelaxation response (pEC_50_). Statistical analysis was performed using either unpaired *t*-tests or by 1-way analysis of variance (ANOVA) with post hoc tests applied as appropriate and as stated in the text (GraphPad Prism, Version 5, Graphpad Software Incorporated). *P* < 0.05 was considered statistically significant.

### 2.10. Drugs and Reagents Used

All drugs and reagents were purchased from Sigma-Aldrich (St. Louis, MO, USA). All drugs were dissolved in dH_2_O, with the exception of LKM, which was dissolved in methanol.

## 3. Results

### 3.1. Systolic Blood Pressure Measurements

**Figure 1:**
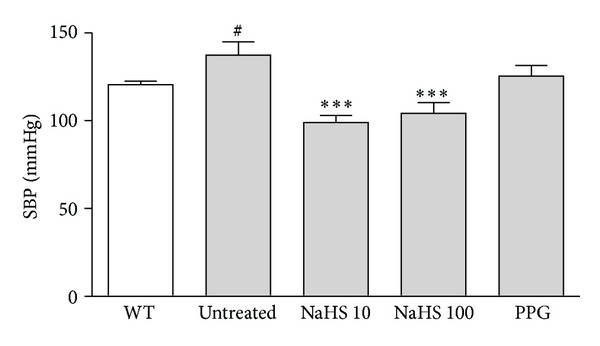
Systolic blood pressure (SBP) measured by the tail-cuff method in WT (open bar) and fat-fed ApoE^−/−^ mice (shaded bar). Fat-fed ApoE^−/−^ mice had a significantly higher SBP than age matched WT controls. SBP was significantly reduced in fat-fed ApoE^−/−^ mice treated with either 10 or 100 *μ*mol/kg/day NaHS. Treatment with PPG 30 mg/kg/day had no effect on SBP. ^#^ *P* < 0.05, *t*-test WT versus fat-fed ApoE^−/−^ mice, ***P < 0.001, ANOVA, untreated versus treated, *n* = 6–9.

Systolic blood pressure measurements were recorded from fat-fed ApoE^−/−^ mice in each treatment group at weeks 12 and 16 (before and after treatment) and in age-matched WT controls at 16 weeks. Measurements collected before treatment showed no significant difference between groups. Fat-fed ApoE^−/−^ mice had significantly higher systolic blood pressure than the age-matched WT controls. Fat-fed ApoE^−/−^ mice treated with either 10 or 100 *μ*mol/kg/day NaHS had a significant reduction in systolic pressure compared to untreated mice (Figure 1). PPG treatment had no significant effect on systolic blood pressure.

### 3.2. Plasma Lipid Levels

Plasma lipid profiles were collected from fat-fed ApoE^−/−^ mice in each treatment group and also from an age matched WT control group. High-fat-fed ApoE^−/−^ mice had significantly greater total cholesterol, and LDL levels when compared to WT controls (Table 1). There was no difference in HDL or plasma triglyceride levels across all groups. Treatment with either 10 or 100 *μ*mol/kg/day NaHS had no effect on plasma lipid profile nor did treatment with PPG (30 mg/kg/day).

### 3.3. Assessment of Atherosclerotic Lesion Area

**Figure 2:**
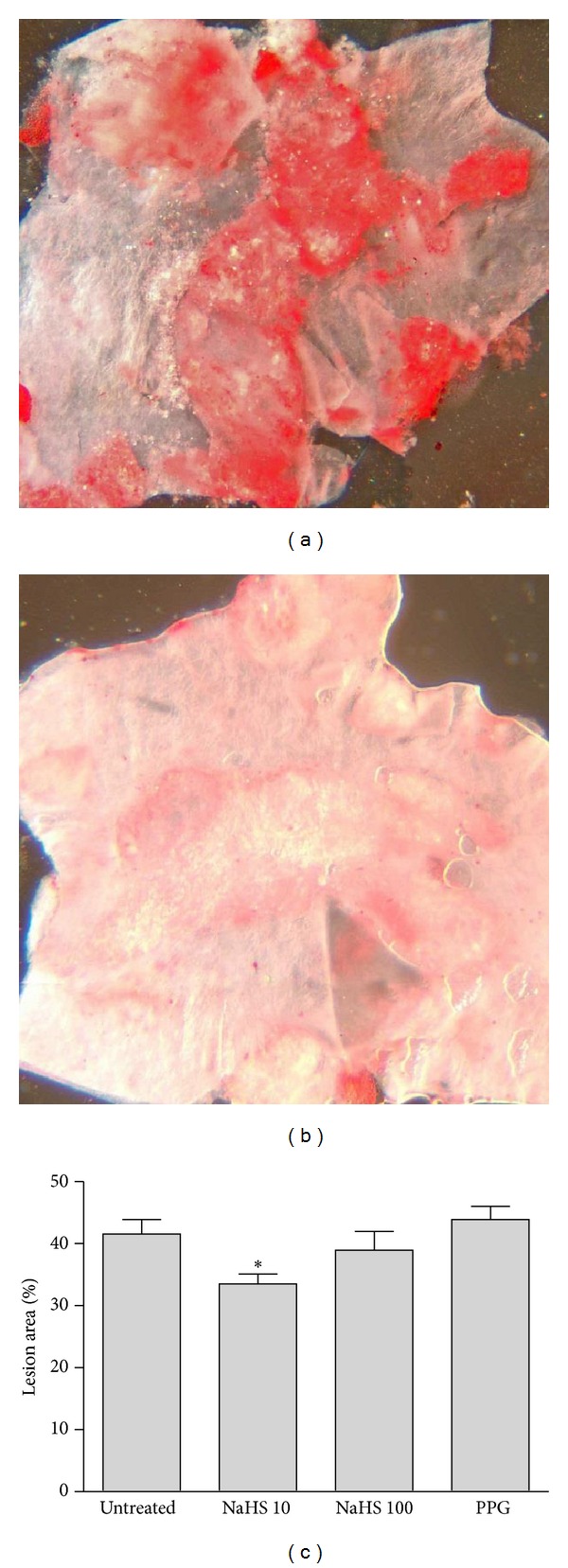
Representative sections of aorta from (a) untreated and (b) NaHS (10 *μ*mol/kg/day) treated fat-fed ApoE^−/−^ mice. (c) Group data for analysis of total lesion area in fat-fed ApoE^−/−^ mice chronically treated with NaHS 10 or 100 *μ*mol/kg/day or PPG 30 mg/kg/day. *P < 0.05, ANOVA, untreated versus treated, *n* = 9–12.

Atherosclerotic lesion development was assessed using *en face* oil red O staining. The luminal surfaces of the aortic arches from the high-fat-fed ApoE^−/−^ mice in each treatment group were examined for total atherosclerotic lesion area. Mice treated with NaHS (10 *μ*mol/kg/day) showed a significant 8% decrease in lesion area when compared with the untreated high-fat-fed ApoE^−/−^ mice. Treatment with PPG had no significant effect on total lesion area (Figure 2).

### 3.4. Assessment of Endothelial Function

**Figure 3:**
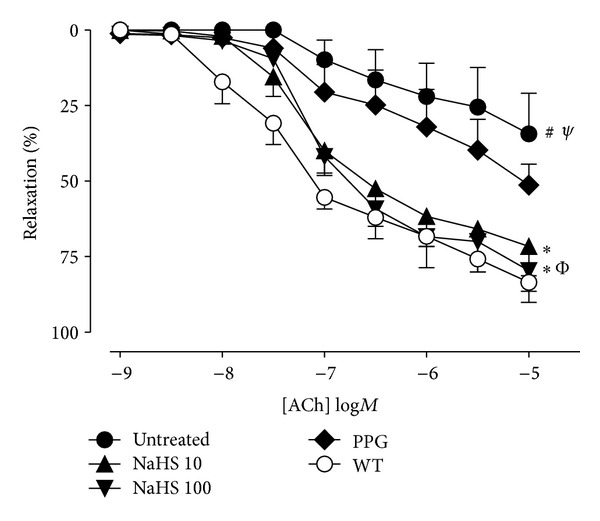
Concentration-response curves to the endothelium-dependent vasodilator acetylcholine (ACh) in aorta from WT (○) and fat-fed ApoE^−/−^ mice. ApoE^−/−^ mice are untreated (●) or chronically treated with 10 (▲) or 100 (*▾*) *μ*mol/kg/day NaHS or PPG 30 mg/kg/day (*◆*). Untreated fat-fed ApoE^−/−^ mice had significantly attenuated response to ACh (*R*_max⁡_ and EC_50_) (^#, Ψ^ *P* < 0.05, *t*-test, WT versus fat-fed ApoE^−/−^). Fat-fed ApoE^−/−^ treated with NaHS at either dose had improved relaxation responses (*R*_max⁡_ and EC_50_) to ACh (^∗, Φ^ *P* < 0.05, ANOVA untreated, versus treated), *n* = 5–7.

Isolated abdominal aorta from high-fat-fed ApoE^−/−^ mice displayed marked endothelial dysfunction compared to WT mice as sensitivity and maximum vasorelaxation responses (*R*_max⁡_) to the endothelium-dependent dilator ACh were significantly impaired (Table 2, Figure 3). Chronic treatment with either dose of NaHS significantly improved endothelial function compared to untreated fat-fed ApoE^−/−^ mice, as both *R*_max⁡_ and pEC_50_ values for these groups were significantly improved and comparable to WT controls. The group treated with PPG had a suppressed *R*_max⁡_ and also displayed impairment in sensitivity to ACh which was similar to that in untreated fat-fed ApoE^−/−^. Relaxation responses to the NO donor sodium nitroprusside (SNP 10 *μ*M) were maximal and of equivalent sensitivity in all treatment groups (data not shown). 

### 3.5. Vascular Superoxide Anion Generation

**Figure 4:**
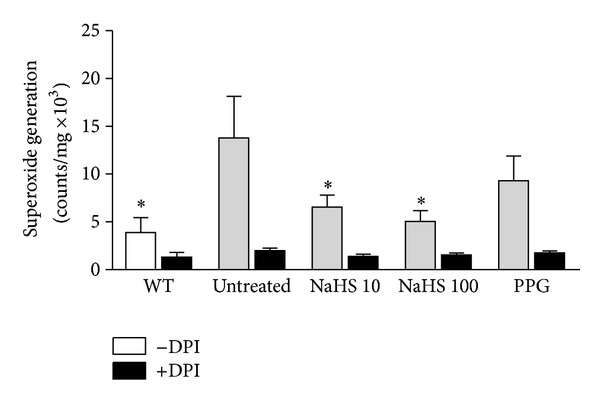
Superoxide generation in aorta from WT control (open bar) and fat-fed ApoE^−/−^ mice (shaded bar) treated with either NaHS (10 or 100 *μ*mol/kg/day) or PPG (30 mg/kg/day). Vascular superoxide generation was significantly increased in fat-fed ApoE^−/−^ mice (^#^ *P* < 0.05, *t*-test, WT versus ApoE^−/−^, *n* = 7). Vascular superoxide production was significantly impaired in the groups treated with either 10 or 100 *μ*mol/kg/day NaHS. (*P < 0.05, ANOVA, versus untreated group, *n* = 7). PPG treatment had no effect. The flavoprotein inhibitor DPI, used to inhibit NADPH oxidase, virtually abolished the vascular superoxide signal (black bars).

Aortic rings from WT and ApoE^−/−^ mice from each treatment group were collected for determination of vascular superoxide generation. Fat-fed ApoE^−/−^ mice had significantly greater vascular superoxide production than age-matched controls. There was a significant inhibition of superoxide production in the 10 and 100 *μ*mol/kg/day NaHS-treated groups (Figure 4). PPG treatment had no significant effect on vascular superoxide generation.

## 4. Discussion

The primary finding of this study is that chronic NaHS treatment *in vivo* has a vasoprotective effect in mice under conditions of high-fat diet and genetic dyslipidaemia. This is the first study to show that chronic exogenous NaHS treatment *in vivo* can cause a reduction in vascular superoxide anion generation, which has the effect of protecting the endothelium from damage thereby preserving endothelial function. In addition, these data show the benefits of NaHS treatment also extend to inhibiting the development of vascular lesions and reducing systolic blood pressure.

Hydrogen sulfide has emerged as an important cardiovascular mediator that has been shown to modulate vascular tone and blood pressure. Deficiency of endogenous H_2_S is reported to play a role in the development of hypertension in spontaneously hypertensive rats [7] and CSE-deficient mice are reported to be hypertensive [5]. The reduction in systolic blood pressure seen in the present study is modest, but significant, and likely results from the vasorelaxant effects of NaHS. These have been attributed primarily to actions at K_ATP_ channels [31], but other K^+^ channel subtypes [8, 23, 26, 32], Ca^2+^ channels [33] and other mechanisms involving PDE and [34] PKG [35] are also implicated.

A most important finding from this study is that chronic NaHS treatment elicits protection of endothelial function. Endothelium dependent vasorelaxation is significantly impaired in ApoE^−/−^ mice fed a high-fat diet, with both sensitivity and maximal relaxation significantly attenuated. Indeed, endothelial dysfunction is a feature of early blood vessel disease and occurs before the development of atherosclerotic lesions. Chronic treatment with NaHS restores endothelial function to the same level as in the wild-type mice. This probably indicates a protection of NO bioavailability since acetylcholine initiated endothelium-dependent vasorelaxation is primarily mediated by NO in mouse aorta [36] and interestingly, the protective effects of NaHS on endothelial function occur at the same dose as the inhibition of vascular superoxide production, suggesting a link between these two events, and consistent with the fact that increased superoxide is known to inhibit NO bioavailability.

A key aspect of the biology of H_2_S is its anti-oxidant effects. H_2_S is a potent one-electron chemical reductant and nucleophile that is theoretically capable of scavenging free radicals by single electron or hydrogen atom transfer [37]. Thus, H_2_S may participate in many reactions [38] and is reported to readily scavenge reactive oxygen and nitrogen species such as peroxynitrite [39], superoxide [40], hydrogen peroxide [41], hypochlorous acid [42] and lipid hydroperoxides [37]. Additionally it has been demonstrated that H_2_S can inhibit the activity and expression of NADPH-oxidase [16], the major vascular source of superoxide. 

It is well known that reactive oxygen species contribute to the pathogenesis of cardiovascular diseases [43]. The parent reactive oxygen species is the free radical superoxide produced by several oxidases including NADPH oxidase, xanthine oxidase, cyclooxygenase and endothelial nitric oxide synthase (eNOS) in its uncoupled state [44]. Overproduction of reactive oxygen species, in particular superoxide from NADPH oxidase, is implicated as a key mediator of endothelial dysfunction and loss of NO bioavailability is associated with many cardiovascular diseases, including diabetic vascular disease, hypertension and atherosclerosis [45]. That oxidative stress apparent in atherosclerosis was affirmed in this study as the fat-fed ApoE^−/−^ mice had significantly greater vascular superoxide generating capacity than the wild-type controls. The lucigenin-based assay employed here showed that the presence of the flavoprotein inhibitor DPI, used to inhibit NADPH oxidase, almost abolished the vascular superoxide production, strongly suggesting that the source of superoxide was an NADPH oxidase. Previous work has shown that NaHS inhibits activity and expression of NADPH-oxidase [16, 46] in cultured vascular smooth muscle cells however the present study extends this finding suggesting an *in vivo* effect of NADPH oxidase inhibition by chronic NaHS treatment and offers a likely molecular mechanism for the vasculoprotective effect of chronic NaHS treatment in this study. 

NaHS treatment had no effect on the plasma lipid profile of these mice. This is not surprising as the fat feeding regime used provides a gross excess of dietary cholesterol. In this model of atherosclerosis, advanced lesions covering nearly 40% of the lumen of the aorta were observed in mice fat-fed for 16 weeks. This is in line with previous studies using this model [47–49]. These vascular lesions were substantially reduced with NaHS treatment for the final 4 weeks of fat feeding, suggesting that the treatment may not just inhibit the progression of lesion development but may also cause regression of lesions. This effect is possibly related to the inhibition of inflammatory responses involving NF-*κ*B and adhesion molecule expression [18]. Additionally, this effect may be due to suppression of vascular superoxide production, since previous studies in this model show a link between atheroprotection and reduced vascular superoxide with consequent increased NO bioavailability [48–50]. 

D,L-propargylglycine is a widely used inhibitor of CSE, despite quite poor selectivity and cell permeability [51]. The dose of PPG used in this study is the same as that used previously in ApoE^−/−^ mice [18] and is maximal as further increases in dose can elicit nephrotoxicity [52]. In the current study CSE activity in the liver, the richest tissue source of the enzyme, was shown to be markedly suppressed in the PPG-treated group (data not shown), suggesting that vascular CSE would also be suppressed, although we did not test this directly. On the basis of using PPG as an inhibitor of CSE, endogenous H_2_S is reported to be involved in the regulation of basal blood vessel tone [8] and indeed blood pressure [7]. Previous studies examining the progression of atherosclerosis in ApoE^−/−^ mice found that CSE activity and endogenous H_2_S production were inhibited in this model [18], results that concur with other data showing an inverse relationship between plasma H_2_S levels and cardiovascular disease. Furthermore Wang et al. [18] found that treatment with PPG exacerbated the atherogenesis in their ApoE^−/−^ mouse model. The present results are in some contrast to this. PPG did not have any exacerbating effect, suggesting that CSE-derived H_2_S does not ameliorate the deleterious effects of the combination of the atherogenic diet and genetic dyslipidemia on blood pressure or vascular dysfunction in this model of atherosclerosis. A simple explanation for this is that endogenous H_2_S would be overwhelmed by the atherogenic insult of the model, which adds a high-fat diet to the genetic dyslipidaemia, a significant enhancement of the atherogenic potency. It is also possible that other non-CSE sources of H_2_S, are upregulated under conditions of CSE inhibition, to replace CSE-derived H_2_S these may include H_2_S derived from cystathionine-beta synthase or 3-mercaptopyruvate transferase and further studies would be required to investigate this possibility.

More work is still required to examine the pharmacokinetics of NaHS and the distribution, sequestration, and metabolism of H_2_S donated from any of the reported H_2_S donor compounds. It is noted that the present results are obtained from a single daily ip dose of NaHS for a relatively short period. NaHS rapidly forms H_2_S when dissolved [53], and it has been shown that intravenously administered NaHS is rapidly removed from the plasma [54]. H_2_S is rapidly consumed in oxygenated tissues [55], so it was not expected that plasma concentrations of sulfide would be different between groups. Plasma H_2_S levels were not measured in this study since we reasoned that they were unlikely to be different between groups and there is controversy over the methods available for doing this [55]. Despite this, previous studies indicate that treatment with similar doses of NaHS in ApoE^−/−^ mice does indeed increase plasma H_2_S levels [18]. That aside, in this study the administered NaHS certainly causes an acute effect which then has longer-term consequences. There is evidence that sulfide donors can cause sulfhydration of specific proteins [56], and though speculative, this is a potential mechanism that would be the starting point for further investigation. 

In the present study a dose dependence of NaHS effect is not apparent for any of the parameters investigated. The beneficial effects of NaHS in reducing blood pressure, improved endothelial function, decreased lesion area and vascular superoxide production were all observed at a dose of 10 *μ*mol/kg/day and this effect was preserved but not enhanced at 100 *μ*mol/kg/day for the SBP data, endothelial function data, and reduced vascular superoxide production, suggesting that the effects on blood vessel tone, endothelial protection, and vascular superoxide production may be linked. These data also indicate that there is a tight therapeutic window for the effects of a sulfide donor in this condition, an observation that is supported by several previous studies (see [57]). The field of H_2_S biology is plagued with a lack of tools, in particular selective and specific blockers of H_2_S-producing enzymes, consistent and stable donors and reliable scavengers. Improvements in these will be most useful for advances in the study of the physiological and pathophysiological effects of H_2_S.

In conclusion, this is the first study to show in an *in vivo* model an inhibition of vascular superoxide generation with chronic NaHS treatment. This effectively protects endothelial function from the oxidative stress induced by the atherogenic diet and renders the vessels resistant to the development of atherosclerotic lesions. An additional beneficial effect of chronic NaHS treatment is a reduction in systolic blood pressure, probably due to vasorelaxant effects. In conclusion, these data show that NaHS donors may be a useful prevention and treatment for vessel damage caused by oxidative stress.

## Figures and Tables

**Table 1:** Effect of chronic treatment with NaHS or PPG on plasma lipid profile.

Group	*n*	Total cholesterol (mM)	HDL (mM)	LDL (mM)	Trigylceride (mM)
WT control	3–5	3.1 ± 0.03	1.3 ± 0.2	0.2 ± 0.02	2.2 ± 0.2
Fat-fed ApoE^−/−^					
Untreated	4–6	32.1 ± 5.9***	2.5 ± 0.4	6.5 ± 1.0***	2.0 ± 0.4
NaHS (10 *μ*mol/kg/day)	4–7	32.3 ± 3.4***	2.4 ± 0.2	7.9 ± 1.0***	1.9 ± 0.3
NaHS (100 *μ*mol/kg/day)	6-7	37.7 ± 2.2***	1.6 ± 0.1	6.9 ± 0.4***	1.9 ± 0.2
PPG 30 mg/kg/day	3–6	33.1 ± 3.2***	2.4 ± 0.5	8.3 ± 1.1***	2.4 ± 0.3

***P < 0.0001, ANOVA, C57 compared with untreated fat-fed ApoE^−/−^.

**Table 2:** Effect of chronic treatment with NaHS or PPG on endothelial function.

Group	*n*	pEC_50_	*R*_max⁡_ (%)
WT control	7	7.36 ± 0.17	84 ± 3
Fat-fed ApoE^−/−^			
Untreated	5	6.36 ± 0.31^Ψ^	34 ± 13^#^
NaHS (10 *μ*mol/kg/day)	7	6.97 ± 0.15*	72 ± 10^Φ^
NaHS (100 *μ*mol/kg/day)	7	6.98 ± 0.06*	78 ± 10^Φ^
PPG (30 mg/kg/day)	5	6.66 ± 0.28	58 ± 9

^#,Ψ^ *P* < 0.05, *t*-test, WT versus fat-fed ApoE^−/−^, ^∗,Φ^ *P* < 0.05, ANOVA, untreated versus treated.
